# Supplementary material for: High-Resolution Microscopic Characterization of Tunneling Nanotubes in Living U87 MG and LN229 Glioblastoma Cells
Source: Cells. 2024 Mar 6;13(5):464. doi: 10.3390/cells13050464 (PMC10931022; doi:10.3390/cells13050464)
Supplement: Supplementary file 1 [file cells-13-00464-s001.zip › cells-2880209-supplementary.pdf]

Supplementary Material

High-Resolution Microscopic Characterization of Tunneling Nanotubes in Living U87 MG and LN229 Glioblastoma Cells

Nicole Matejka[1*], Asieh Amarlou[1], Jessica Neubauer[1], Sarah Rudigkeit[1] and Judith Reindl[1]

[1]Institute for Applied Physics and Measurement Technology, University of the Bundeswehr Munich, Neubiberg, Germany

*** Correspondence:** Nicole Matejka: Nicole.matejka@unibw.de

1 Supplementary Tables

Dye	Used WLL intensity	SNR	Background
CellMask™ Orange Plasma Membrane	5 % - 15 %	5.7 ± 3.4	11.3 ± 5.7
CellMask™ Green Plasma Membrane	5 % - 15 %	5.7 ± 2.9	6.8 ± 2.4
DiO	30 %	2.5 ± 0.8	14.1 ± 5.6
PKH	10 % - 20 %	1.7 ± 0.8	14.5 ± 4.5
WGA	20 % - 30 %	0.72 ± 0.4	34.5 ± 9.7
CellLight™	85 %	0.8 ± 0.6	13.7 ± 2.6
MemGlow™	10 % - 30 %	4.8 ± 3.5	18.5 ± 3.3

Table S1: Used White Light Laser (WLL) intensity for the microscopy of U87 cells labeled with different stains. The measured Signal-to-Noise ratio (SNR) for TNTs and the measured Background gray value for each stain used. Means ± standard deviations are shown.

Cell line	Mean length ± SD [µm]	Mean lifetime ± SD [min]	Nummer of measured TNTs	Treatment (experiment)
U87 MG	41 ± 26	81 ± 58	66	Non-treated (1)

U87 MG	42 ± 25	87 ± 48	51	4 Gy X-ray irradiation (1)
U87 MG	48 ± 36	87 ± 68	66	Non-treated (2)
U87 MG	49 ± 31	98 ± 64	51	4 Gy X-ray irradiation (2)
LN229	20 ± 11	44 ± 27	46	Non-treated
LN229	20 ± 12	37 ± 32	39	4 Gy X-ray irradiation

Table S2: Measured lengths and lifetimes of TNTs in untreated and 4 Gy X-ray treated U87 MG and LN229 cells from all independent experiments. Means ± standard deviations are shown.

2 Supplementary Figures

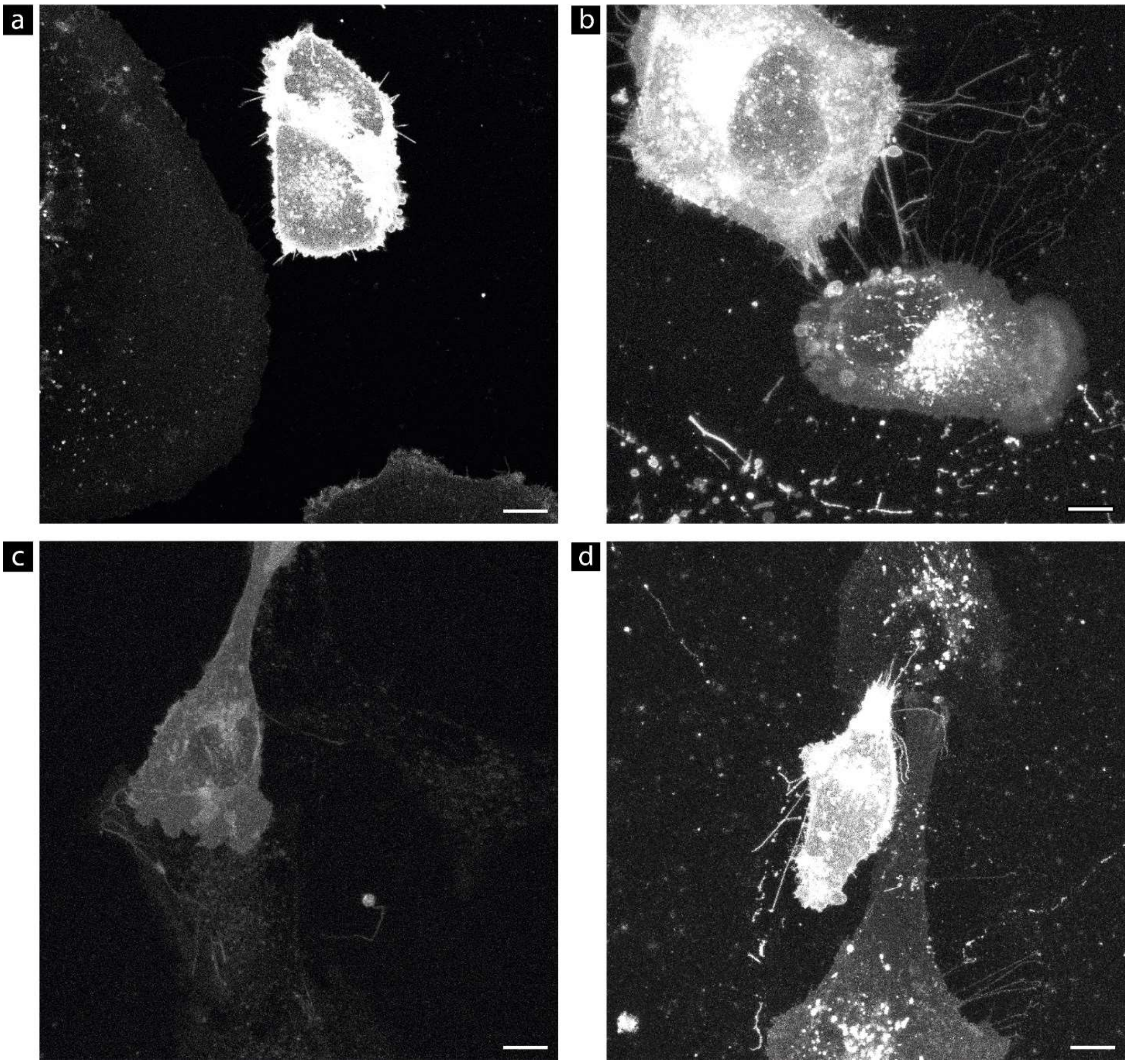

Figure S1: Inhomogeneous labeling of U87 cells using DiO (a), PKH (b), CellLight™ (c) and MemGlow™ (d). All images are maximum projections. Brightness and contrast were not adjusted. Scale bar: 10 µm.

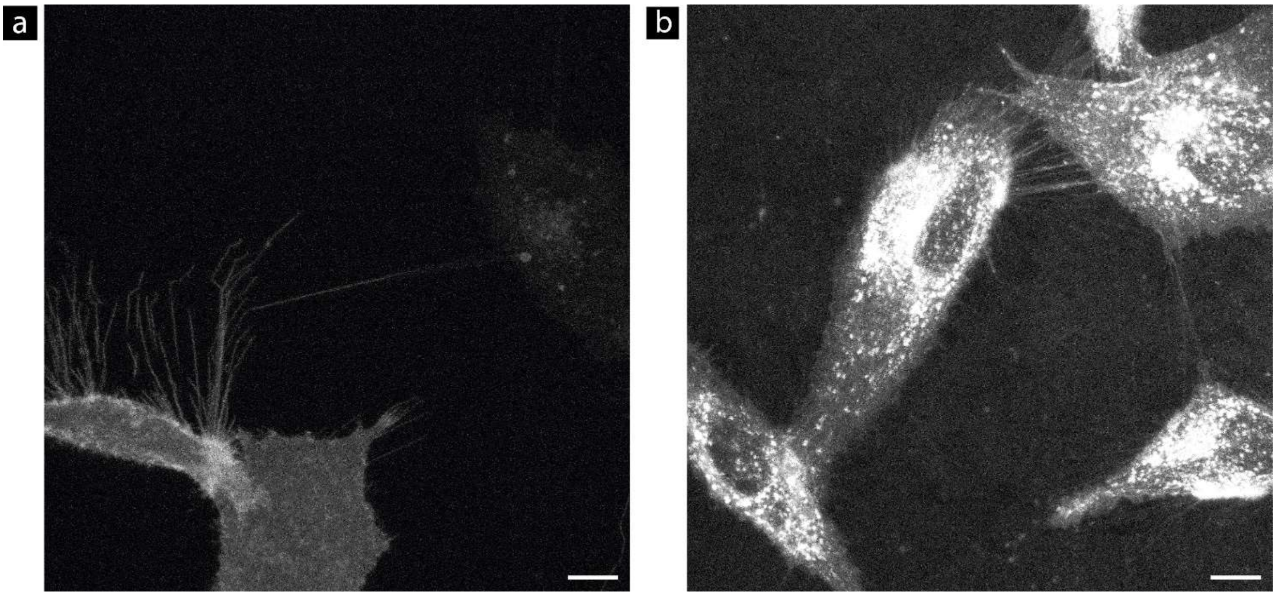

Figure S2: Poor SNR for CellLight™ (a) and WGA (b). Due to the low signal yield of CellLight™ a high WLL intensity of 85% was necessary and even at this high intensity some cells are not well visible like the cell in the upper right corner of (a). Also, labeling with WGA results in very punctate labeling of the membrane with high background noise. All images are maximal projections. U87 cells are shown. Brightness and contrast have not been adjusted. Scale bar: 10 µm.

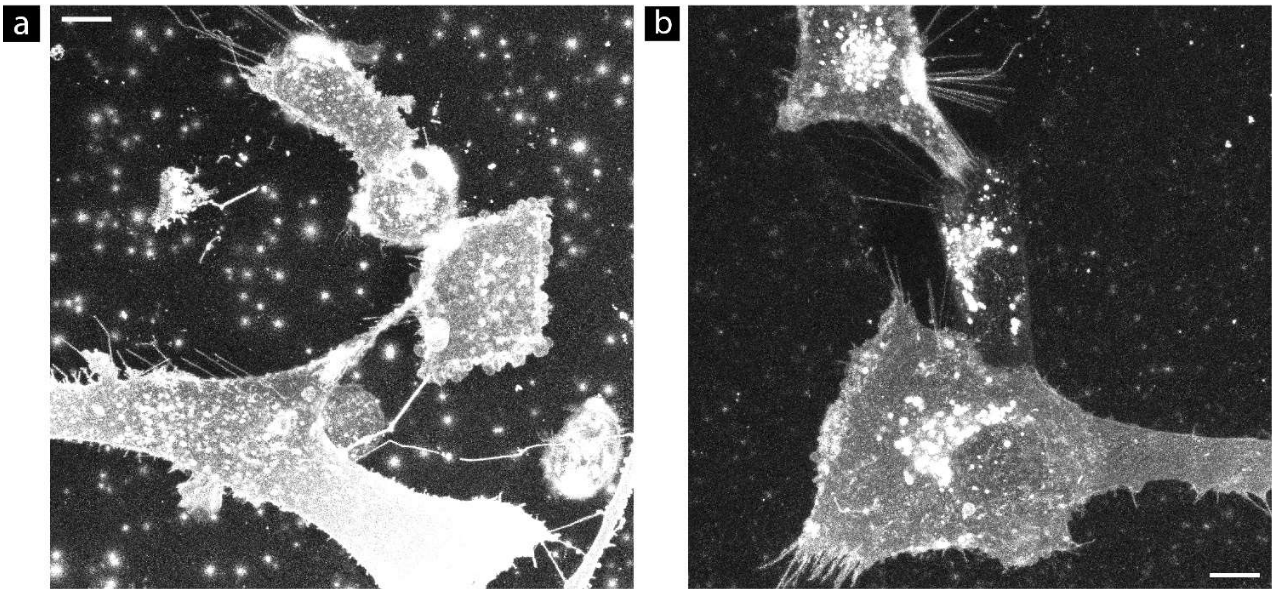

Figure S3: Accumulation of MemGlow™ dye on the glass bottom. In (a) the U87 cells were labeled with serum-free media and in (b) with normal growth media. The accumulation of the dye is better when normal growth media is used as loading buffer. However, the labeling is not homogeneous when using normal growth media. All images are maximum projections. Brightness and contrast have not been adjusted. Scale bar: 10 µm.

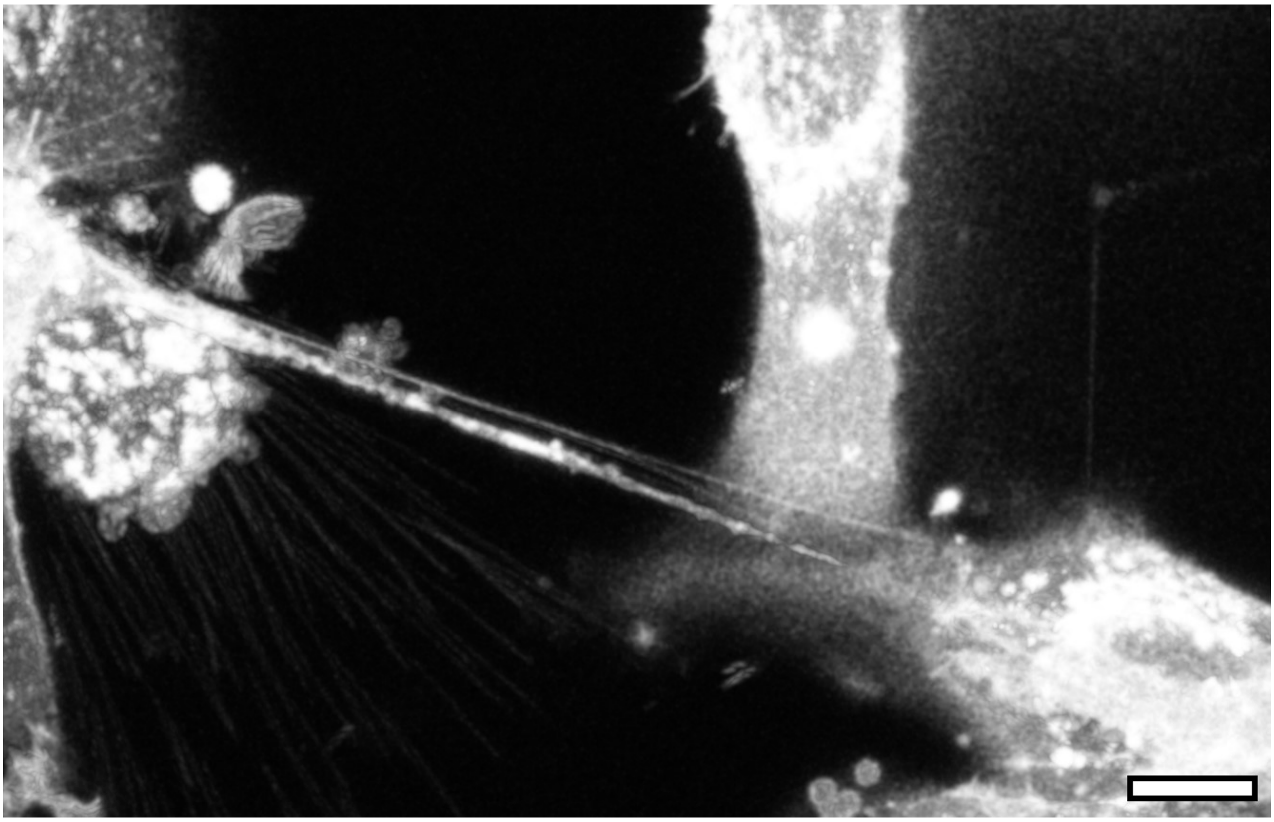

Figure S4: TNTs can only be distinguished from EP bridges by their diameter. Two cells are connected by a TNT and also by an EP bridge. Shown are U87 cells labeled with CellMask™ Orange Plasma Membrane. The image is a maximum projection. Scale bar: 10 µm.

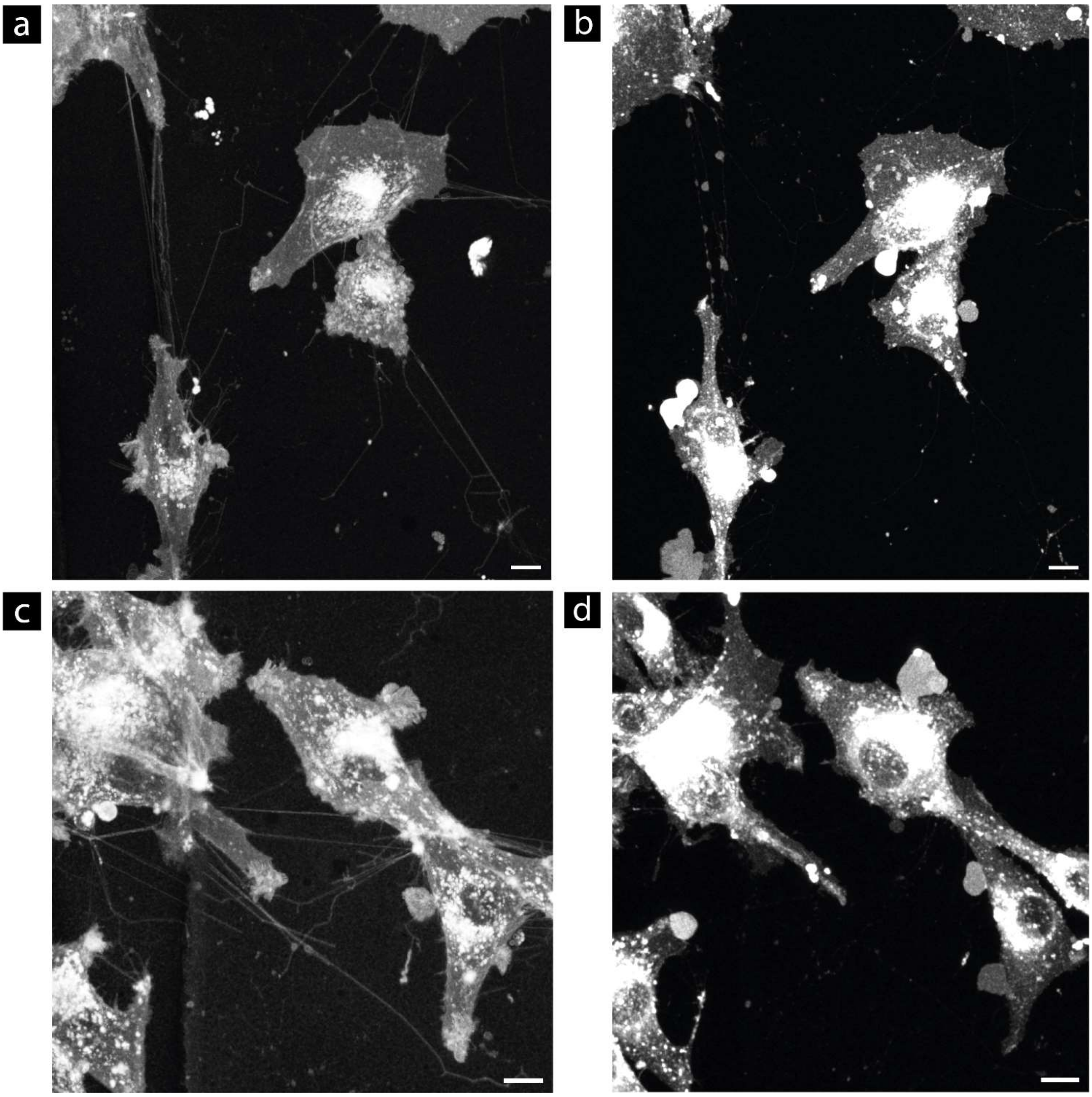

Figure S5: Loss of TNTs after fixation with formaldehyde. Shown are live-cell images (a and c) of membrane-labeled U87 cells and acquired images of the same cells after fixation (b and d). Shown are exemplary images of sample 1 (a and b) and sample 2 (c and d) from one experiment. Images are maximum projections. U87 cells were labeled with CellMask™ Orange Plasma Membrane. Scale bar: 10 µm.

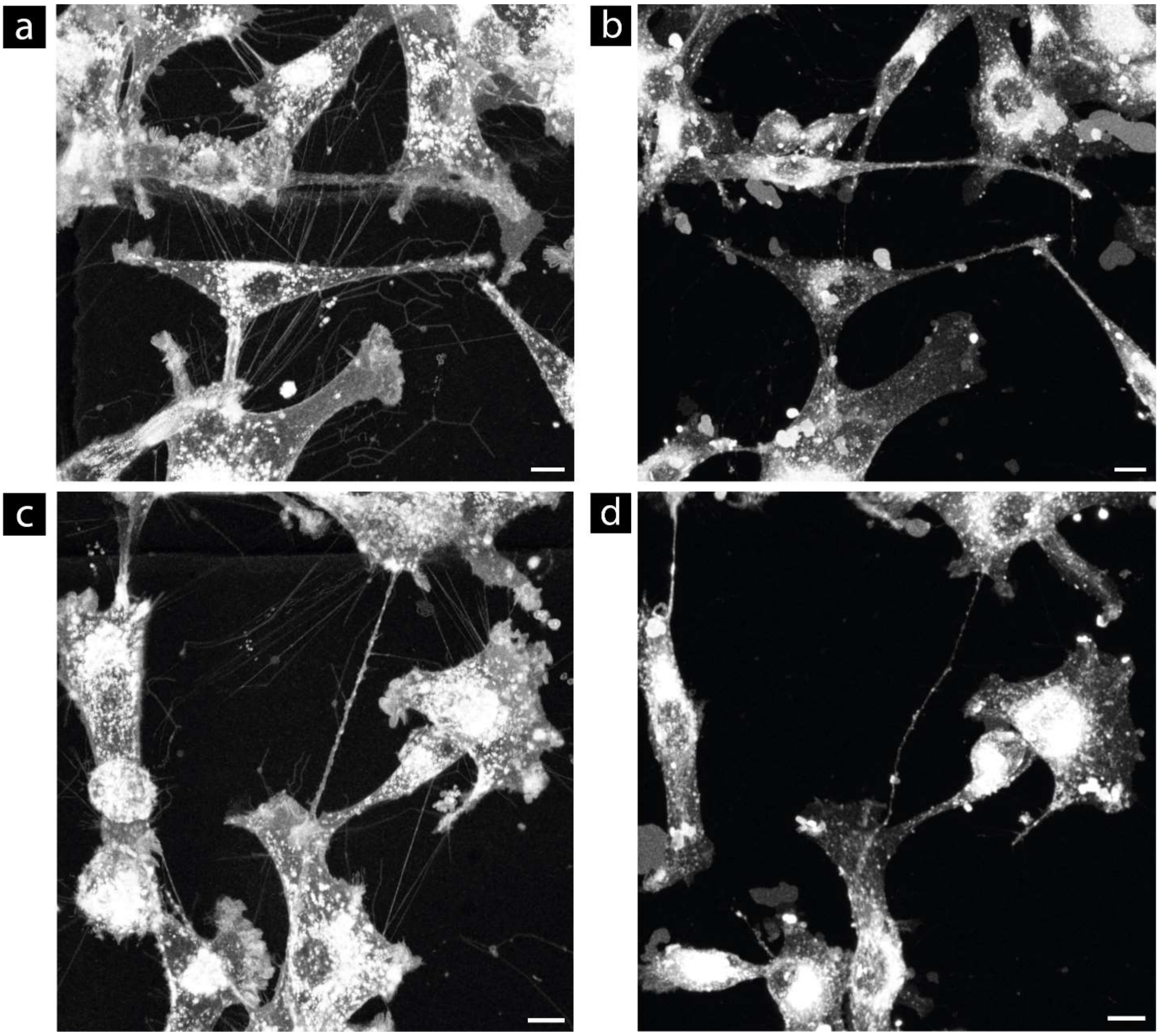

Figure S6: Loss of TNTs after fixation with formaldehyde. Shown are live-cell images (a and c) of membrane-labeled U87 cells and acquired images of the same cells after fixation (b and d). Shown are exemplary images of sample 3 (a and b) and sample 4 (c and d) from one experiment. Images are maximum projections. U87 cells were labeled with CellMask™ Orange Plasma Membrane. Scale bar: 10 µm.
